# Structure and Function of the Bi-Directional Bacterial Flagellar Motor

**DOI:** 10.3390/biom4010217

**Published:** 2014-02-18

**Authors:** Yusuke V. Morimoto, Tohru Minamino

**Affiliations:** 1 Quantitative Biology Center, RIKEN, 6-2-3 Furuedai, Suita, Osaka 565-0874, Japan; E-Mail: ymorimoto@fbs.osaka-u.ac.jp; 2 Graduate School of Frontier Biosciences, Osaka University 1-3 Yamadaoka, Suita, Osaka 565-0871, Japan

**Keywords:** Bacterial flagellum, rotary motor, motility, stator, rotor, torque generation, mechanosensor

## Abstract

The bacterial flagellum is a locomotive organelle that propels the bacterial cell body in liquid environments. The flagellum is a supramolecular complex composed of about 30 different proteins and consists of at least three parts: a rotary motor, a universal joint, and a helical filament. The flagellar motor of *Escherichia coli* and *Salmonella enterica* is powered by an inward-directed electrochemical potential difference of protons across the cytoplasmic membrane. The flagellar motor consists of a rotor made of FliF, FliG, FliM and FliN and a dozen stators consisting of MotA and MotB. FliG, FliM and FliN also act as a molecular switch, enabling the motor to spin in both counterclockwise and clockwise directions. Each stator is anchored to the peptidoglycan layer through the *C*-terminal periplasmic domain of MotB and acts as a proton channel to couple the proton flow through the channel with torque generation. Highly conserved charged residues at the rotor–stator interface are required not only for torque generation but also for stator assembly around the rotor. In this review, we will summarize our current understanding of the structure and function of the proton-driven bacterial flagellar motor.

## 1. Introduction

**Figure 1:**
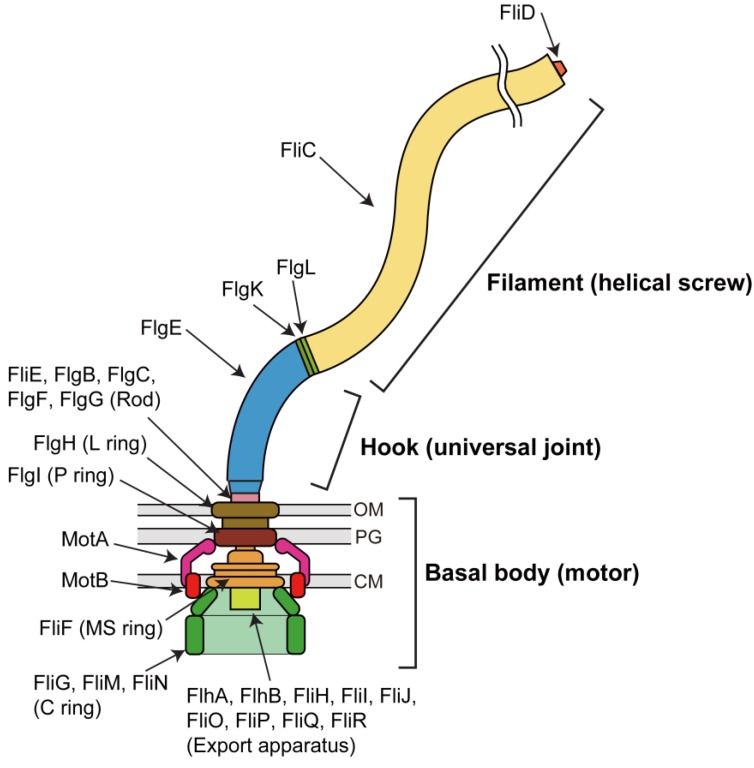
Schematic diagram of the bacterial flagellum. The flagellum is a locomotive organelle for bacterial propulsion. The flagellum consists of the basal body, which acts as a reversible rotary motor, the hook, which functions as a universal joint and the filament, which works as a helical screw. OM, outer membrane; PG, peptidoglycan layer; CM, cytoplasmic membrane.

Many bacteria swim in liquid media by rotating bacterial flagella. The bacterial flagellum is a supramolecular complex made of about 30 different proteins with copy numbers ranging from a few to a few tens of thousands (Figure 1). The flagellum consists of at least three parts: the basal body, the hook and the filament. The basal body is embedded within the cell membranes and acts as a rotary motor. The hook and filament extend outwards in the cell exterior. The filament works as a helical screw to produce the thrust. The hook exists between the basal body and filament and functions as a universal joint to smoothly transmit torque produced by the motor to the helical filament [1,2,3]. 

The flagellar motor of *Escherichia coli* and *Salmonella enterica* is fueled by the electrochemical potential of protons (H^+^) across the cytoplasmic membrane, i.e., the proton motive force [1,3,4,5]. The flagellar motor can operate in either a counter-clockwise (CCW, viewed from filament to motor), or clockwise (CW) direction. When most of the motors rotate in the CCW direction, the filaments form a bundle and propel the cell smoothly. When one or more motors spin in the CW direction, the bundle is disrupted and hence the cell tumbles and changes the swimming direction [6,7]. *E. coli* and *Salmonella* can move towards more favorable conditions and escape from undesirable ones for their survival by sensing temporal variations of environmental stimuli, such as chemical attractants and repellents, temperature and pH via methyl-accepting chemotaxis proteins (MCP). MCPs are transmembrane proteins with a large cytoplasmic domain involved in interactions with a histidine kinase CheA, and an adaptor protein CheW. The MCPs control CheA autophosphorylation. Phosphorylated CheA transfers its phosphate group to a response regulator CheY. Phosphorylated CheY (CheY-P) binds to the cytoplasmic face of the flagellar motor, allowing the motor to spin in the CW direction [8]. 

**Figure 2:**
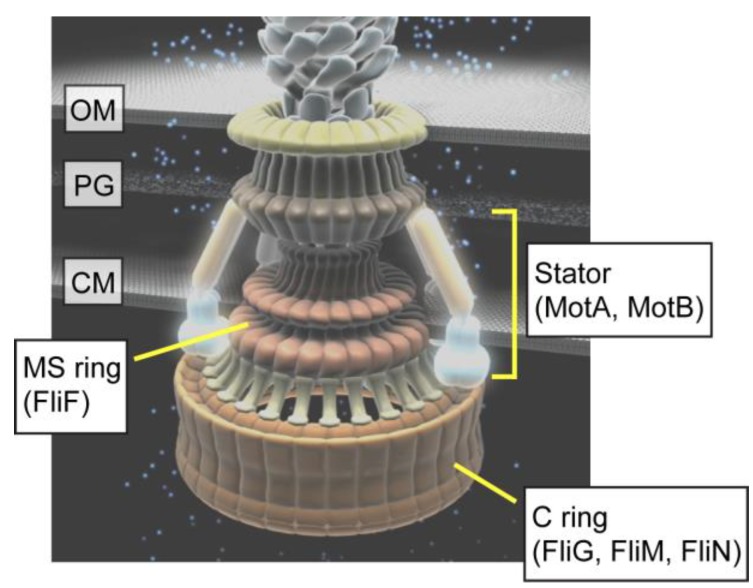
Schematic diagram of the proton-driven bacterial flagellar motor. The flagellar motor consists of a reversible rotor made of FliF, FliG, FliM and FliN and a dozen stators, each of which consists of MotA and MotB. FliF forms the MS ring within the cytoplasmic membrane. FliG, FliM and FliN form the C ring on the cytoplasmic face of the MS ring. OM, outer membrane; PG, peptidoglycan layer; CM, cytoplasmic membrane.

The flagellar motor consists of a rotor and a dozen stators (Figure 2). The rotor consists of four flagellar proteins, FliF, FliG, FliM and FliN. FliF forms the MS ring within the cytoplasmic membrane [9,10]. FliG, FliM and FliN form the C ring on the cytoplasmic face of the MS ring [11,12]. FliG, FliM and FliN are also responsible for switching the direction of motor rotation, enabling the motor to rotate in either CCW or CW [13]. Each stator consists of two integral membrane proteins, MotA and MotB [14,15,16] and is postulated to be anchored to the peptidoglycan (PG) layer through a well-conserved peptidoglycan binding (PGB) motif within the C-terminal periplasmic domain of MotB (MotB_C_) [17]. MotA and MotB form a proton channel complex to couple proton flow through the channel with torque generation [18,19,20,21]. Torque is generated by interactions between MotA and FliG [22]. This article covers our current discoveries of the proton-driven flagellar motor of *E. coli* and *Salmonella* with the emphasis being on the structure, function and dynamic properties of the motor. 

## 2. Rotor

The rotor consists of two ring structures, the MS and C rings (Figure 2). The MS ring is formed by 26 copies of FliF and acts as an assembly platform [10]. Using the MS ring as a template, FliG, FliM and FliN assemble into the C ring onto the cytoplasmic face of the MS ring [12]. FliG directly associates with the cytoplasmic face of the MS ring with a 1 FliG : 1 FliF stoichiometry [23,24], suggesting that the FliG ring has the 26-fold symmetry. FliG consists of three domains, FliG_N_, FliG_M_ and FliG_C_ [25]. FliG_N_ directly binds to the MS ring [26,27]. FliG_C_ is directly involved in the interaction with a stator protein MotA [22]. This suggests that FliG_C_ is located at the upper part of the C ring. FliG_M_ provides a binding site for FliM [28,29,30,31]. FliM and FliN form a stable complex with a 1 FliM: 4 FliN stoichiometry and occupy most part of the C ring [32]. The C ring has rotational symmetry that varies from 32-fold to 36-fold [33], and the diameter actually shows a range of variability (45 ~ 49.0 nm) [34]. Crystal structures of FliG [25,35,36,37], FliM [38], FliN [32,39] and the FliG-FliM complex [29,30,31] have been solved, and possible models for their organization have been proposed. Please see a recent review article focusing on the current models of the rotor ring structure [40]. 

Certain mutations in FliG, FliM and FliN affect the switching between CW and CCW rotation, indicating that these three proteins are responsible for switching the direction of motor rotation [13]. CheY-P binds to FliM and FliN, inducing cooperative conformational changes of the FliG ring to allow the motor to spin in the CW direction [41,42]. The switching between the CW and CCW states is highly cooperative [43]. The cooperative switching mechanism can be explained by a conformational spread model, in which a switching event is mediated by conformational changes in a ring of subunits that spread from subunit to subunit via nearest-neighbor interactions [44,45]. When motor speed is increased up to 100–150 Hz, both the CW-to-CCW and CCW-to-CW switching rates increase linearly and then decrease with speed to an equivalent degree, without changing the motor bias [46]. This suggests that the motor switch can sense the load on the stator–rotor interaction as well as the cytoplasmic level of CheY-P [46]. A mathematical model to explain this load-switching relationship of the motor has been proposed [47]. Recently, it has been reported that the nucleotide second messenger cyclic di-GMP (c-di-GMP), which promotes transition of single motile cells to surface-attached multicellular biofilm, regulates flagella-driven motility. A c-di-GMP binding protein YcgR binds to MotA, FliG and FliM in the presence of c-di-GMP, resulting not only in a significant reduction in motor speed but also in the CCW bias in the motor rotation [48,49,50].

Mutations located in and around helix_MC_, which connects FliG_M_ and FliG_C_, generate a diversity of phenotype, including motors that are strongly CW biased, infrequent switchers, rapid switchers, and transiently or permanently paused. This suggests that helix_MC_ is involved in switching of the direction of flagellar motor rotation [51]. The *Salmonella* FliG(ΔPAA) mutant motor, which has three-amino-acid deletion at positions 169 to 171, are extremely CW biased [52]. This mutant motor remains in the CW rotation even in the absence of CheY-P, indicating that the motor is locked in the CW state [37]. This suggests that the PAA deletion induces a conformational change of FliG at the rotor–stator interface in a way similar to the binding of CheY-P to FliM and FliN. Limited proteolysis has shown that the PAA deletion results in a conformational change in a hinge between FliG_M_ and helix_MC_. This result is in agreement with the crystal structure of *Thermotoga maritima* FliG_MC_(ΔPEV) showing that the orientation of helix_MC_ relative to FliG_M_ is distinct from that seen in the wild-type *T. maritima* FliG_MC_ structure [36,37]. However, it remains unclear how such a change in the orientation of helix_MC_ relative to FliG_M_ affects the switching process. 

The flagellar motor containing a single stator unit rotates with 26 steps per revolution, which is in agreement with the stoichiometry of the FliG ring [53,54]. The elementary process of torque generation by stator–rotor interactions is symmetric in CCW and CW rotation [54], although the torque-speed curves are distinct between them [55]. To achieve the symmetric elementary process of torque generation in both CCW and CW rotation, FliG_C_ may need to go through a 180º rotation to reorient its charged residues in the two opposite directions. However, it remains unknown how it occurs. 

FliM-GFP (or YPet) and GFP-FliN show turnovers between the motor and their cytoplasmic pools [56,57], indicating that the C ring is a highly dynamic structure rather than a static one. The turnover of FliM occurs only in the presence of CheY-P, suggesting that this turnover is directly involved in the switching process. The number of FliM subunits in the C ring increases in response to a reduction in the concentration of CheY-P, suggesting that the flagellar motor adapts to changes in the steady-state level of CheY-P by adjusting the number of FliM molecules to which CheY-P binds [58,59].

## 3. Stator

### 3.1. Proton Channel of the MotA/B Complex

**Figure 3:**
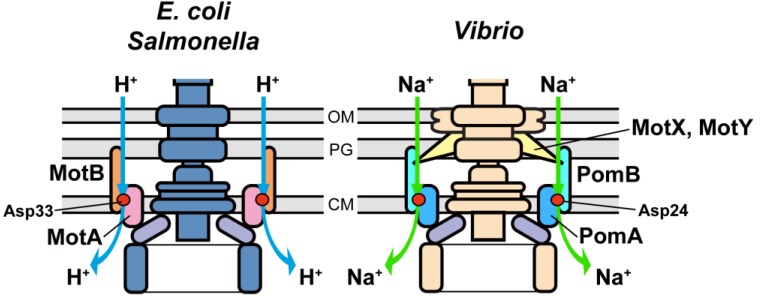
Schematic diagrams of the bacterial H^+^-driven and Na^+^-driven flagellar motors. *E. coli* and *Salmonella* have the H^+^-driven flagellar motor (left) whereas *Vibrio alginolyticus* has the Na^+^-driven polar flagellar motor (right). The stator of the flagellar motor consists of MotA and MotB in the H^+^-driven motor and of PomA and PomB in the Na^+^-driven motor. MotX and MotY are required for efficient assembly of the PomA/B complex around the motor in *Vibrio*.

**Figure 4:**
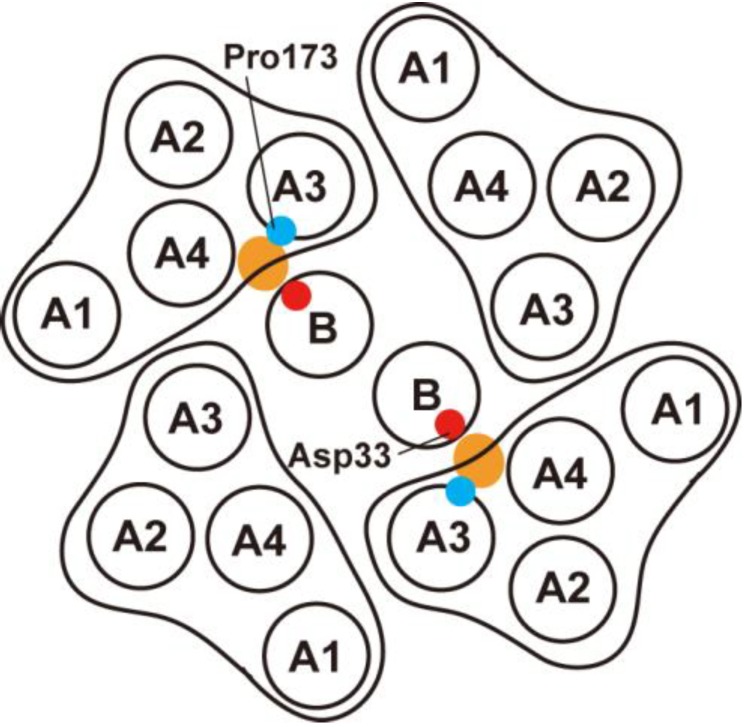
Arrangement of transmembrane segments of the MotA/B complex, which consists of four copies of MotA and two copies of MotB. The view is from the periplasmic side of the membrane. The complex has two proton conducting pathway shown by orange ellipsoids [79,80,81].

The energy for flagellar motor rotation is supplied by the electrochemical potential of specific ions across the cytoplasmic membrane [60,61,62,63]. Based on the coupling ions, the flagellar motors are divided into at least two types (Figure 3): one is the H^+^-driven type found in *E. coli* and *Salmonella* [18,21,52,65,66] and the other is the Na^+^-driven type in marine *Vibrio* and extremely alkalophilic *Bacillus* [67,68,69]. Recently, it has been reported that extremely alkaliphilic *Bacillus* *alcalophilus* Vedder 1934 utilizes both Na^+^ and K^+^ as the coupling ions for flagellar rotation [64]. The stator of the H^+^-driven motor consists of four copies of MotA and two copies of MotB [70] and acts as a proton channel to couple proton flow through the channel with torque generation (Figure 4) [18]. Terahara *et al.* [71] have reported that the MotA/B complex of alkaliphilic *Bacillus clausii* can conduct bothH^+^ and Na^+^ at different pH ranges. The Na^+^-coupled stator complex of *Vibrio alginolyticus* consists of four proteins, PomA, PomB, MotX and MotY [72]. PomA and PomB form a Na^+^-conducting pathway within the cytoplasmic membrane in a way similar to the MotA/B proton channel [73]. MotX and MotY form the T ring located beneath the P ring for efficient assembly and stable anchoring of the PomA/B complex to its binding site on the motor [74]. Interestingly, both H^+^-coupled and Na^+^-coupled stators are incorporated into a single flagellar motor in *Bacillus subtilis* and *Shewanella oneidensis* MR-1 [75,76]. Please see a review article summarizing the current understanding of the Na^+^-driven flagellar motor in *Vibrio alginolyticus* [72]. 

MotA consists of four transmembrane spans (TMs), two short periplasmic loops and two extensive cytoplasmic regions. MotB consists of an N-terminal cytoplasmic region, one TM and MotB_C_ containing a well-conserved PGB motif. Asp-33 of *Salmonella* MotB, which corresponds to Asp-32 of *E. coli* MotB, is a highly conserved aspartic residue [66,77]. This aspartic acid residue is located at the proximal end of MotB-TM and is critical for proton translocation through the channel [52,65,66]. MotB exists as a homo-dimer in the stator and these aspartic acid residues are positioned on the surface of the MotB-TM dimer facing MotA-TMs [78]. These observations suggest that the MotA/B complex has two proton-conducting pathways (Figure 4). MotA-TM3 and MotA-TM4 are close to MotB-TM while MotA-TM1 and MotA-TM2 are in the outer positions of the MotA/B complex [79]. This suggests that MotB-TM forms a proton channel along with the TM-3 and TM-4 helices of MotA (Figure 4). The V35F mutation, which lies in MotA-TM2, has been isolated as an extragenic suppressor of the *motB(D33E)* mutant [80]. It is impossible for the side chain of Val-35 in MotA-TM2 to directly access the proton-conducting pathway formed at the interface between MotA and MotB because it is likely to be located within the hydrophobic core of the four transmembrane α-helix bundle of MotA. This suggests that TM2 is presumably close to TM3 and TM4 to maintain the proton pathway. This is supported by site-directed disulfide cross-linking experiments [81].

Pro-173 of MotA, which is highly conserved among MotA orthologues, is located at the proximal end of MotA-TM3 (Figure 4) [82,83]. Site-directed disulfide cross-linking experiments have shown that Pro-173 of *E. coli* MotA is in relative close proximity to Asp-32 of *E. coli* MotB [81]. Co-overproduction of MotA with the MotB-TetA chimera results in proton leakage, arresting cell growth [18]. The *E. coli* MotB(D32N) mutation suppresses the proton leakage through the MotA/B-TetA proton channel. Interestingly, introduction of the MotA(P173D) mutation into the MotA/B(D32N)-TetA complex causes massive proton flow again, largely impairing the cell growth. This indicates that this Asp-173 residue of MotA allows protons to go through the channel in the absence of Asp-32 of MotB, probably by presenting a carboxyl residue as an alternative proton-binding site [83]. These observations suggest that Pro-173 of MotA forms a part of the proton-conducting pathway along with Asp-32 of MotB. The *Salmonella* MotA(P173A) mutant motor produces torque at the wild-type level when the motor operates under a very high load condition [84]. However, the MotA(P173A) mutation causes ca. 90% reduction in the maximum rotation rate, suggesting that the P173A mutation significantly reduces the rate of the mechanochemical reaction cycle of the motor [84]. Simulation of the torque-speed curve of the MotA(P173A) mutant motor by a simple kinetic model indicates that the P173A mutation slows the rates of conformational changes of the MotA/B complex by 25 times [84]. Because a prolyl residue induces bending in TM to facilitate ion transport [85], Pro-173 of MotA is proposed to facilitate the conformational changes of the MotA/B complex that support the rapid mechanocmemical cycle when the motor rotates at high speed [81,84]. 

Over-expression of the MotA/B complex from a plasmid does not affect cell growth at all. In contrast, in-frame deletion of a plug segment formed by residues 53 to 66 in MotB causes massive proton flow through a proton channel, thereby arresting cell growth [21,86]. This suggests that the plug segment interferes with proton channel formation, thereby suppressing undesirable proton flow through the channel when the MotA/B complex is not installed into the motor. Interestingly, however, the cell growth is not significantly impaired by in-frame deletion of residues 51 to 100 in *Salmonella* MotB, which contains the plug segment although MotB(∆51–100) is partially functional [87,88]. This indicates that proton channel formation of the MotA/B(∆51–100) complex does not occur prior to its assembly around the motor although the plug is missing. The L119P and L119E substitutions allow unassembled MotA/B(∆51–100) complex to conduct protons to a significant degree, impairing the cell growth [21,88]. This suggests that not only the plug segment but also some other region within MotB_C_ regulate proper proton channel formation.

### 3.2. Role of MotB_C_ in Stator Assembly around a Rotor

**Figure 5:**
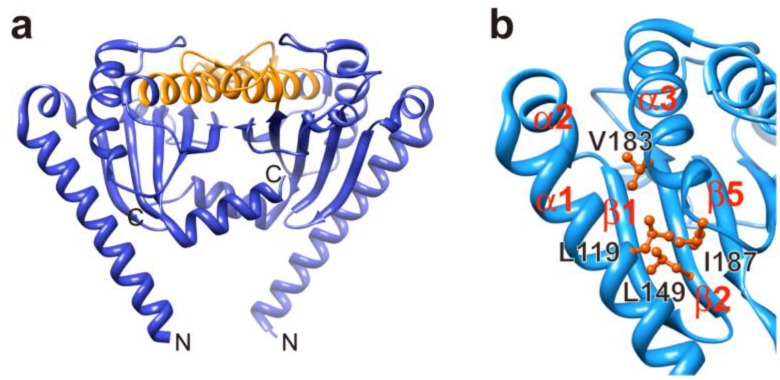
The crystal structure of MotB_C_ from *Salmonella*. (**a**) Cα ribbon representation of *Salmonella* MotB_C_ dimer in the crystal (PDB ID: 2ZVY). The peptidoglycan binding (PGB) motif is shown by orange. (**b**) The MotB_C_ structure in the vicinity of Leu119. Leu119 in helix α1 shows hydrophobic interactions with Leu149 in strand β2 and Val183 and Ile187 after helix α3.

MotB_C_ forms a homo-dimer and its dimerization is required for MotB function [88,89]. MotB_C_ is required not only for proper anchoring of the stator to the PG layer [90] but also for proper alignment of the stator relative to the rotor [91]. The crystal structure of the core domain of MotB_C_, which consists of residues 99 to 276 in MotB, shows considerable structural similarities with the PGB domain of Pal (Figure 5a) [88]. Interestingly, a chimeric MotB-Pal protein, in which the PGB domain of E. coli MotB is replaced by that of E. coli Pal, exerts MotB function to a considerable degree, suggesting that MotB_C_ binds to the PG layer through its PGB motif in a way similar to Pal [92]. Site-directed disulfide crosslinking experiments have shown that MotB_C_ is in relatively close proximity to the P ring of the basal body [93].

Upon induction of the MotA/B complex from a plasmid in a *motAB* null mutant, a stepwise increment in speed is observed at high load, estimating that there are as many as 11 stators around the rotor [96]. Interestingly, abrupt, stepwise drops and restorations of the rotation speed of the motor are also observed even in steadily rotating motors, indicating that the stators are replaced frequently [14,15,53]. Consistently, GFP-fused MotB shows rapid exchanges between the motor and the cytoplasmic membrane pool, indicating that an interaction between MotB_C_ and the PG layer is highly dynamic [97]. These observations suggest that the MotA/B complexes are no permanently fixed in place around the motor during motor rotation. 

**Figure 6:**
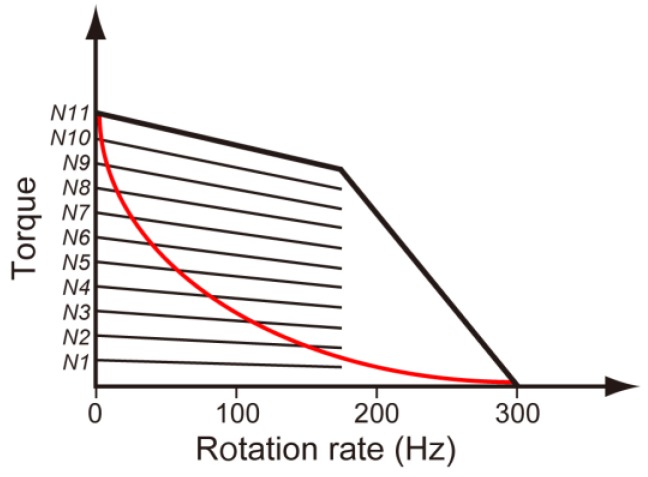
Torque-speed curves of the wild-type (black line) and MotB(∆72–100) mutant motors (red line). Torque-speed curves of the wild-type motor with different number of stator units are indicated (*N*_1_–*N_11_*).

The torque-speed curve of the flagellar motor consists of two regimes: a high-load, low-speed regime and a low-load, high-speed regime (Figure 6). In the high-load, low-speed regime, the speed of the motor is proportional to the number of stators, whereas in the low-load, high-speed regime, one stator unit can spin the motor at the maximum speed [94,95]. Recently, it has been reported that a few stators spin the motor at low loads and that 6–11 stators drive the same motor at high loads. This suggests that the flagellar motor controls the number of functional stators in the motor in response to changes in external load [98,99]. In contrast to the torque-speed curve of the wild-type motor with a gradual decrease in the high-load regime and a rapid drop in the low-load regime, in-frame deletion of 72 to 100 in MotB causes an unusual torque-speed curve of the motor with a rapid drop in the high-load regime and a slow decrease in the low-load regime (Figure 6) [100]. At a low speed near stall, the MotB(∆72–100) mutant motor produces torque at the wild-type level, indicating that the deletion does not affect an actual torque-generation step by stator-rotor interactions coupled with proton translocation through the proton channel. In contrast, as motor speed increases, torque produced by the MotB(∆72–100) mutant motor drops off at much higher load than that of the wild-type motor due to a rapid decrease in the number of stators driving the motor at much higher load than that the wild-type motor responds to dissociate its stator from the motor [100]. As torque generation by the motor applies an equal and opposite force on the PG layer through MotB_C_, MotB_C_ is proposed to act as a load-sensitive structural switch to regulate the assembly and disassembly cycle of the stators in response to the load changes and that an appropriate length of a linker connecting the PGB domain to MotB-TM may stabilize this structural switch during the torque generation cycle [100].

### 3.3. Load-Sensitive Coupling between Proton Translocation and Torque Generation

Both protonation and deprotonation of MotB-Asp33 induce conformational changes of the cytoplasmic loop between TM2 and TM3 of MotA (MotA_C_), which is directly involved in the interaction with FliG. This suggests that proton translocation through the proton channel is coupled with cyclic conformational changes of the MotA/B complex for torque generation [101]. The MotB(D33E) mutation in MotB causes a slow motile phenotype [52]. This mutation results in about 50% reduction in near-stall torque. Interestingly, the suppressor mutations, which exist in MotA-TM2, MotA-TM3, MotB-TM and MotB_C_, restore stall torque to the wild-type level [80]. The D33E mutation does not decrease the number of stators around the rotor, indicating that the decrease in stall torque is not due to the reduction in the number of stators driving the motor [102]. Since the rotation rate of the flagellar motor is not limited by the rate of the mechanochemical cycle of the motor at low speed near stall [103], it is suggested that the D33E mutation misaligns MotA_C_ relative to FliG at the rotor–stator interface, causing the 50% reduction in the energy coupling efficiency of the motor and that the suppressor mutations readjusts the alignment of MotA_C_ relative to FliG, thereby restoring the coupling efficiency to the wild-type level [102].

The MotB(D33E) mutation causes a 90% reduction in the maximum rotation speed of the flagellar motor at a very low load, indicating that this mutation results in a considerable reduction in the maximum rate of the mechanochemical cycle of the motor. The suppressor mutations do not support the maximal rotation rate at low load [80]. Because the proton conductivity of the unplugged proton channels of the MotA/B(D33E) and its suppressor MotA(V35F)/B(D33E) complexes are measured to be ca. 50% and ca. 60% of that through unplugged wild-type channel, the decreased proton conductivity through the channel itself is not the main cause of the marked reduction in the maximal rotation speed at low load [102]. The rate of proton flow through the MotA/B proton channel is limited by the rate of conformational change of the MotA/B complex, suggesting that the 90% reduction in the maximal rotation speed of these mutant motors is due to a considerable decrease in rates of the conformational changes of the MotA/B complex coupled with proton translocation through the channel [102].

The stator has a high duty ratio and hence remains attached to the rotor for most of the mechanochemical reaction cycle [94,95]. The rotation speed of the wild-type motor is very stable over a wide range of rotation rates [95]. However, the rotation speeds of the MotA/B(D33E) and MotA(V35F)/B(D33E) motors show large fluctuations at low load although they are stable at high load. Interestingly, frequent pauses are also observed for these two mutant motors at low load [102]. These observations suggest that the wild-type MotA/B complex can sense even a small load to continue the mechanochemical reaction cycle for torque generation by efficiently coupling proton translocation with its conformational changes. Because the stators are dynamic mechanosensors and change their structure to control the number of stators driving the motor in response to changes in external load [98,99], it is proposed that the MotA/B complex is a load-sensitive proton channel that efficiently couples proton translocation with torque generation in response to changes in the load and that MotB-Asp33 is important for this coordinated proton translocation [102]. 

## 4. Role of Stator-Rotor Interactions in Efficient Assembly of the Stators into the Motor

**Figure 7:**
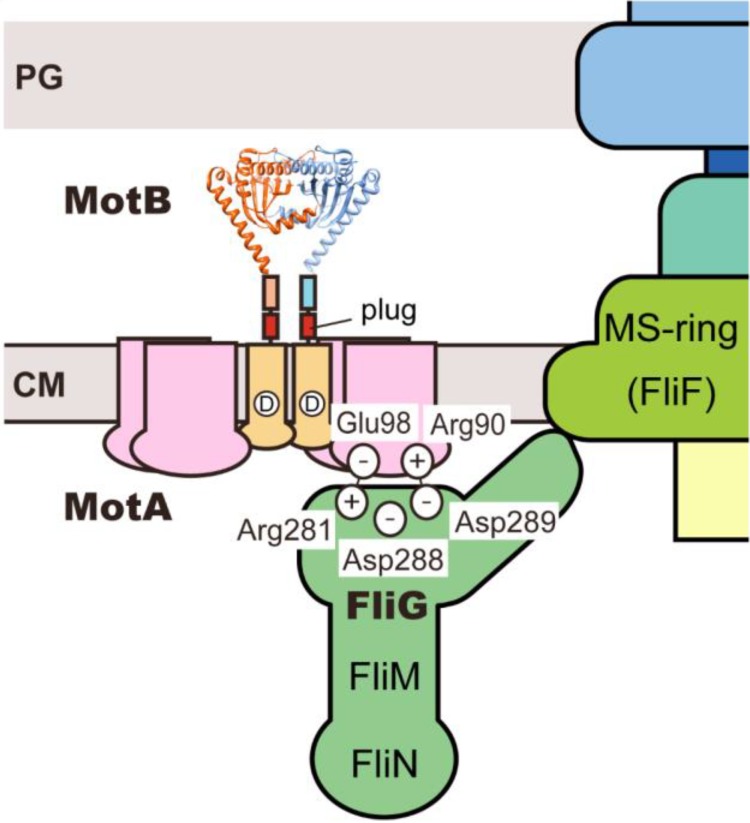
Schematic diagram of interactions at the rotor–stator interface. FliG is postulated to occupy the upper part of the C ring. The interactions between MotA-Arg90 and FliG-Asp289 and between MotA-Glu98 and FliG-Arg281 are directly involved not only in torque generation but also in stator assembly around the rotor.

MotA-Arg90 and MotA-Glu98, which are located in MotA_C_, are highly conserved residues among MotA orthologues and are critical for motor function [104]. The extragenic FliG(D289A) and FliG(D289K) suppressor mutations partially rescue the motility of the *motA(R90E)* mutant whereas the extragenic FliG(R281V) and FliG(R281W) suppressor mutations restore the motility of the *motA(E98K)* mutant, indicating genetic interactions between MotA-Arg90 and FliG-Asp289 and between MotA-Glu98 and FliG-Arg281 (Figure 7) [22]. This suggests that these interactions at the rotor–stator interface are directly involved in flagellar rotation. The MotA(R90E), MotA(E98K), FliG(D289K) and FliG(R281V) mutations considerably affect the assembly of GFP-labeled MotA/B complexes around the rotor, suggesting that these interactions are also critical for stator assembly around the motor [105,106]. The FliG(D289K) mutation significantly recovers the localization of GFP-MotB to the motor in the *motA(R90E)* mutant whereas the FliG(R281V) mutation does not recover the GFP-MotB localization in the *motA(E98K)* mutant. These suggest that the MotA-Arg90−FliG-Asp289 interaction is critical for the proper positioning of the stators around the rotor, whereas the MotA-Glu98−FliG-Arg281 interaction is more important for torque generation [106].

*Salmonella* MotB missing residues 51 to 100 retains the ability to form the functional stator with MotA to a considerable degree [87]. The distance between the surface of the hydrophobic core layer of the cytoplasmic membrane and that of the PG layer is about 100 Å. The crystal structure of a *Salmonella* MotB_C_ fragment corresponding to residues 99 to 276 of MotB is only about 50 Å tall [88] and hence it cannot reach the PG layer if connected directly to MotB-TM by the deletion of residues 51–100. Therefore, a large conformational change must be required for the PGB sites on the top surface of MotB_C_ to reach the PG layer. Leu119 in helix α1 shows hydrophobic interactions with Leu149 in strand β2 and Val183 and Ile187 after helix α3 (Figure 5b) [88]. The L119P and L119E mutations, which not only cause massive proton flow through the proton channel of the MotA/B(∆51–100) complex but also increase the assembly efficiency of the MotA/B(∆51–100) complex into the motor, would destabilize these hydrophobic interactions [21,88]. This raises a plausible hypothesis that these hydrophobic interactions act as a structural switch to regulate the assembly and disassembly of the stators [88,100]. Over-expression of MotA inhibits wild-type motility due to a reduction in the number of the functional stator around the rotor [105]. Consistently, MotA-mCherry co-localizes with GFP-FliG even in the absence of MotB [105]. These observations indicate that MotA alone can be installed into the motor. Because conserved charged residues at the rotor–stator interface are directly involved in stator assembly around the motor [105,106], interactions between MotA and FliG may trigger a large conformational change in MotB_C_ that open the proton channel and allow the stator to be anchored to the PG layer.

## 5. Conclusions

Both the rotor and stator of the flagellar motor are highly dynamic structures rather than static ones. [56,57,58,97,98,99] Two rotor proteins, FliM and FliN, show highly dynamic turnovers between the motor and their cytoplasmic pools. The FliM turnover plays an important role in switching the direction of flagellar motor rotation [56,58,59]. However, little is known about the switching mechanism of the rotor ring complex. The stators are machanosensors to regulate the number of the stators driving the motor in response to changes in external load [98,99,100]. Each stator is also a load-sensitive proton channel that efficiently couples proton translocation with torque generation in response to the load changes [102]. However, it remains unknown how the stators sense the amount of external load to control the number of functional stators around the rotor as well as the proton flow through the channel. Interactions at the rotor–stator interface are important, not only for torque generation [22], but also for efficient stator assembly around the rotor [105,106]. However, it remains unknown how the rotor–stator interactions allow the stator to open the proton channel and to be anchored to the PG layer. We need to investigate the switching mechanism of the flagellar motor in much more detail by biophysical and structural techniques combined with genetic and biochemical approaches. Super-resolution fluorescence microscopic techniques would be required for understanding of the mechanosensing mechanism of the stator. Furthermore, not only *in vitro* reconstitution experiments but also high-resolution structural analysis by X-ray crystallography and electron cryomicroscopy would be essential to advance our mechanistic understanding of the energy coupling mechanism of the flagellar motor. 
